# The interaction between permethrin exposure and malaria infection affects the host-seeking behaviour of mosquitoes

**DOI:** 10.1186/s12936-019-2718-x

**Published:** 2019-03-14

**Authors:** Kevin Thiévent, Gaël Hauser, Obada Elaian, Jacob C. Koella

**Affiliations:** Institute of Biology, University of Neuchâtel, Rue Emile-Argand 11, 2000 Neuchâtel, Switzerland

**Keywords:** Insecticide-treated bed nets (ITNs), Irritancy, Sub-lethal effects, Malaria infection, Host-seeking behaviour

## Abstract

**Background:**

Insecticide-treated bed nets (ITNs) help to control malaria by mechanically impeding the biting of mosquitoes, by repelling and irritating them and by killing them. In contrast to spatial repellency, irritancy implies that mosquitoes contact the ITN and are exposed to at least a sub-lethal dose of insecticide, which impedes their further blood-seeking. This would weaken the transmission of malaria, if mosquitoes are infectious.

**Methods:**

It was therefore tested whether sub-lethal exposure to permethrin impedes blood-feeding differently in uninfected mosquitoes and in mosquitoes carrying the non-transmissible stage (oocysts) or the infectious stage (sporozoites) of the malaria parasite *Plasmodium berghei*. In addition, as the degree of irritancy determines the dose of insecticide the mosquitoes may receive, the irritancy to permethrin of infected and uninfected mosquitoes was compared.

**Results:**

In this laboratory setting, sub-lethal exposure to permethrin inhibited the blood-seeking behaviour of *Anopheles gambiae* mosquitoes for almost 48 h. Although infection by malaria did not affect the irritancy of the mosquitoes to permethrin at either the developmental stage or the infectious stage, both stages of infection shortened the duration of inhibition of blood-seeking.

**Conclusions:**

The results suggest that the impact of ITNs may be weaker for malaria-infected than for uninfected mosquitoes. This will help to understand the global impact of ITNs on the transmission of malaria and gives a more complete picture of the effectiveness of that vector control measure.

**Electronic supplementary material:**

The online version of this article (10.1186/s12936-019-2718-x) contains supplementary material, which is available to authorized users.

## Background

In the last few decades, intensifying malaria control with insecticides that target adult mosquitoes, in particular insecticide-treated bed nets (ITNs), has decreased drastically the burden of malaria [1]. The efficacy of ITNs is partly due to their dual mode of protection: they protect the community by killing mosquitoes, thereby lowering the likelihood that mosquitoes live long enough to transmit malaria, and they protect individuals with the physical barrier of the net and the insecticide’s repellency to mosquitoes, thereby reducing the risk that users become infected [2].

The repellency of insecticides has two components: spatial repellency and contact irritancy [3]. Spatial repellency occurs when mosquitoes detect the volatiles of the insecticide and then avoid the area near the ITN. It thus occurs before mosquitoes have had any physical contact with the ITN. Irritancy occurs when the physical contact of the mosquitoes with the insecticide makes them fly away. The relative importance of the two mechanisms of repellency depends on the insecticide. Permethrin, for example, is only slightly spatially repellent but strongly irritant, whereas DTT repels and irritates mosquitoes [3–5].

Since irritancy implies that mosquitoes contact the insecticide, and thus are exposed to at least a small (sub-lethal) dose of the insecticide, one of the differences between irritancy and spatial repellency is that the former may further affect the mosquitoes’ physiology or behaviour [6–8].

Most studies of sub-lethal effects on mosquitoes deal with larval exposure to pesticides and its effects on life-history traits (reproductive success, adult longevity, sex ratio [9–13]) and on vector competence, e.g., arboviruses [14–16] and malaria [17]. While sub-lethal exposure of adults has been less well studied, a general feature of exposure to insecticides, in particular pyrethroids, appears to be that it reduces the feeding motivation for some time after exposure. This has been observed in tsetse flies (*Glossina* spp.) [18, 19], spider mites (*Tetranychus urticae*) [20], *Drosophila melanogaster* [21], *Aedes aegypti* [22], and in several species of *Anopheles* [23]. Sub-lethal exposure to pyrethroids also reduces the time of activation to flight and alters the flight direction of mosquitoes *Culex quinquefasciatus*, *Ae. aegypti* and *A. albimanus* [24]. These effects probably impede the subsequent search for hosts and may explain that the presence of ITNs reduces the number of mosquitoes that re-enter a house immediately after having exited it [25]. Overall, it thus appears that irritant pyrethroid insecticides can protect users of ITNs from being bitten and reduce the feeding motivation of mosquitoes to protect non-users.

Both of these aspects of protection have the greatest impact on malaria transmission when mosquitoes carry sporozoites, since both irritancy and feeding inhibition will then keep people from being infected. Unfortunately, since sporozoites make mosquitoes more motivated to bite [26–30], it could be expected that the insecticide is less protective against infectious mosquitoes. Indeed, in a previous experiment infectious mosquitoes (carrying sporozoites) were less repelled by ITNs than uninfected ones [31]. But how malaria infection affects the irritancy or feeding inhibition caused by pyrethroid insecticides is unknown.

Therefore, with this study, it was investigated whether infection of the mosquito *A. gambiae* by malaria affects the irritancy and the feeding inhibition induced by permethrin, a pyrethroid insecticide. In particular, it was tested whether mosquitoes infected with the non-infectious oocyst stage or the infectious sporozoite-stage of the rodent malaria parasite, *Plasmodium berghei*, are more or less inhibited by sub-lethal exposures to permethrin than uninfected mosquitoes. Although mosquitoes are increasingly resistant against pyrethroids, the experiment was done with the insecticide-sensitive Kisumu colony of *A. gambiae.* The experiment thus gives a first impression of the impact of an insecticide on the feeding behaviour of malaria-infected mosquitoes, and is not an attempt to predict the situation in the field. In particular we do not consider the long-term impact of sub-lethal exposure to an insecticide in resistant mosquitos, which may include impeded parasite development and transmission and cumulative reduction of mosquito survival over time [32]. Our experiment was done with permethrin because of its use on several commercially available ITNs (e.g. Olyset^®^ and Olyset^®^ Plus nets (Sumitomo Chemical Co. Ltd., Tokyo, Japan) [33]).

## Methods

### Mosquito and parasite

The experiment was done with the Kisumu colony of *A. gambiae* from Western Kenya [34] and with a strain of the rodent malaria parasite *P. berghei* ANKA expressing green fluorescent protein (GFP). Professor Heussler from the University of Bern kindly provided mice infected with gametocytes to infect the mosquitoes.

### Mosquito rearing and infection

The tests for irritancy and for sub-lethal effects on feeding motivation were done in two separate experiments. For irritancy 600 mosquito larvae were reared individually in 12-well-plates in 3 ml of de-ionized water, while for the sub-lethal effects 1200 larvae were reared in groups of 200 in trays (35 × 21 × 4 cm) containing 1 l of deionized water. In both experiments, larvae were reared with a standard level of fish food [34] (day of hatching: 0.04 mg of Tetramin fish food per larva; day 1 after hatching: 0.06 mg; age 2: 0.08 mg; age 3: 0.16 mg; age 4: 0.32 mg; age 5 and more: 0.6 mg), pupae were haphazardly moved to cages (21 × 21 × 21 cm) and the males were removed at most 24 h after emergence so that the females were virgin. The females were first maintained at 26 ± 1 °C and 70 ± 5% humidity. One day before infection, the temperature was decreased by 2 °C every 4 h until it reached 19 ± 1 °C, which is the optimal temperature for the development of *P. berghei* in the mosquito [35]. The mosquitoes were given access to a 6% sugar solution throughout the experiment except for the day before the blood meal.

In each experiment half of the 3–4 days old females were blood-fed on gametocytic mice, the other half were blood-fed on uninfected mice. Mice were anesthetized with an intraperitoneal injection of 8.5 ml/kg of a mix of Xylazine Xylasol^®^ (solution: 20 mg/ml), Ketamine Ketasol^®^ (solution: 100 mg/ml) and PBS [36]. They were then placed on the top of the cages and mosquitoes were given the opportunity to blood-feed for 20 min. One day after feeding, the females that were not completely engorged were removed from the cages. The mosquitoes were then maintained at 19 °C and 70% humidity and with constant access to a 6% solution of sugar up to the tests.

### Test for irritancy

Irritancy of the nets was measured for 40 infected and 39 uninfected mosquitoes 11 days after blood feeding (when infected mosquitoes harboured oocysts) and for 72 infected and 64 uninfected mosquitoes 21 days after blood feeding (when most of them harboured sporozoites). The mosquitoes were placed individually into the resting part of WHO test tubes [37]. After 2 min of acclimatization, the mosquitoes were gently blown into the exposure part of the tube containing a 0.75% permethrin-impregnated paper (WHO standard paper). The time until their first jump (the time between landing on the paper and flying in an attempt to escape from the paper [38]) and the number of times they jumped between their first jump and 2 min were then recorded. This gives two independent measures of irritancy. Note that the number of jumps was measured only from the time of the first jump onwards, so that this measure of irritancy would be independent of the first. The mosquitoes were then frozen at − 20 °C until further analysis.

### Test for motivation to blood feed

Seventy-two infected and 80 uninfected mosquitoes were tested 11 days after feeding (when infected mosquitoes harboured oocysts), and 96 infectious and 107 uninfected mosquitoes were tested 21 days after feeding (when they harboured sporozoites). A third of the mosquitoes in each group were exposed either to a control paper (PY control), to 0.75% permethrin-impregnated paper for 1 min or to the permethrin for 2 min. They were placed in groups of six into the resting tube of the WHO susceptibility test tubes. After 2 min, the mosquitoes were gently blown into the test tube. After their exposure, the mosquitoes were transferred individually into 120-ml plastic cups and given the opportunity to feed on a piece of cotton soaked with a 6% sugar solution.

To measure the mosquitoes’ motivation to blood feed, GH placed one of his arms onto each mosquito’s cup repeatedly (2, 9, 24, 30, 36, and 48 h after exposure to the insecticide) until it responded and tried to bite. The mortality of mosquitoes during the trials was recorded. All mosquitoes were frozen after the trials and kept at − 20 °C for further analysis.

### Detection of *Plasmodium berghei* infection

#### Oocyst detection

To detect the oocysts, 11-day old mosquitoes were dissected in 10 µl of a 0.9% NaCl solution and the midguts were examined under a dissecting microscope with a fluorescent filter for the GFP. Infection was recorded as the presence or absence of oocysts.

#### Sporozoite detection

Sporozoites were detected with a PCR of the head and thorax of the 21-day old mosquitoes as described by Thiévent and coworkers [31]. DNA was extracted with DNAzol (MRC Inc., Cinncinati, OH, USA). Head and thorax were smashed with a pellet in 200 μl of DNAzol and were incubated for 20 min at 55 °C. The solution was then centrifuged at 20,000×*g* for 10 min; 170 μl of the supernatant were transferred to a new fresh tube containing 3 μl of PolyAcryl Carrier (MRC Inc., Cincinnati, OH, USA) and 100 μl of 100% ethanol conserved at − 20 °C. After a centrifugation at 15,000×*g* during 8 min, ethanol was discarded. DNA was washed with 0.8 ml of 75% ethanol (− 20 °C) and the tube was centrifuged 5 min at 15,000×*g*. Ethanol was discarded and the pellet of DNA was dried in a Speedvac for 15 min at 45 °C. Finally, the DNA was eluted in 50 μl of de-ionized water.

PCR amplification was done with a T3000 thermocycler (Analytik Jena AG, Jena, Germany) with 3 μl of DNA and 1 μl of a 10 μM solution of each primer [forward (5′-ACGATGATATAGATCAAAT-3′) and reverse (5′-TACCTAAGCTTCTTGCGTA-3′)]. Primers amplify a 111 bp sequence of the merozoite surface protein-1 (MSP-1) gene of *P. berghei* NK65 and ANKA. Amplification was done during 40 cycles with denaturation at 95 °C, annealing at 54 °C and extension at 72 °C, each for 45 s. Infection was visually confirmed by the presence/absence of the correct band of DNA by gel electrophoresis with a 2% agarose gel containing 9 µl of RedSafeTM Nucleic Acid stain (iNtRON Biotechnology, Korea).

### Body size

The body size of mosquitoes was assayed as their wing length. Wings were placed onto a slide, photographed, and measured with the software ImageJ. The length of each wing was measured from the distal end of the alula to the tip of the wing (the end of the vein R3) without the fringes.

### Statistical methods

The contact irritancy of permethrin was assessed by analysing: (i) the time it took mosquitoes to jump off of the insecticide-treated filter paper; and, (ii) the jumping rate, which is the number of jumps per the remaining time after their first jump (Additional file 1: Table S1). The time until first jump was analysed with a survival analysis with a mixed effect Cox model (*coxme* library in R [39]). The jumping rate was log-transformed, so that the distribution was Gaussian, and was analysed with a linear mixed model. Both analyses included the infection status (infected or not) and the time after infection (11 or 21 days) as nominal factors, wing length as a covariate and the mouse on which the mosquito had blood fed as a random factor.

The measure of motivation to bite was the time it took for mosquitoes to start biting after being exposed to permethrin. Because of the design (e.g., the irregular sampling points of 2, 9, 12, 24, 30, 36, 48 h after exposure), an interval count analysis was used instead of a more classical survival analysis (see [40]). Since the data followed neither an exponential nor a Weibull distribution, a cox-hazard survival analysis was approximated by analysing the number of mosquitoes feeding at a given time as a function of the interval of time between two measurements [40] (Additional file 1: Table S2). The exposure (permethrin or control), the infection status (infected or not), the time after infection (11 or 21 days) were added as nominal factors, and wing length as a covariate. To obtain the results shown below, non-significant interactions were removed from the best model (with p-values greater than 0.25). Significance of the effects were assessed using a type 3 anova (*car* library in R [41]).

All analyses and graphs were done with the software R (version 3.4.3) [42] with the Rstudio interface (version 1.1.419) [43]. Mixed models (LMM and GLMM) were done with the *lme 4* library in R [44].

## Results

For the irritancy experiment, 69.6% of the mosquitoes that blood-fed on gametocytic mice were infected, while for the sub-lethal effects experiment, 84.5% of them were infected. The mosquitoes that had fed on infected mice but did not harbour parasites were removed from the analysis. In total, the behaviour of 510 mosquitoes was analysed, of which 88 harboured oocysts, 132 harboured sporozoites and 290 were uninfected.

### Irritancy

**Fig. 1:**
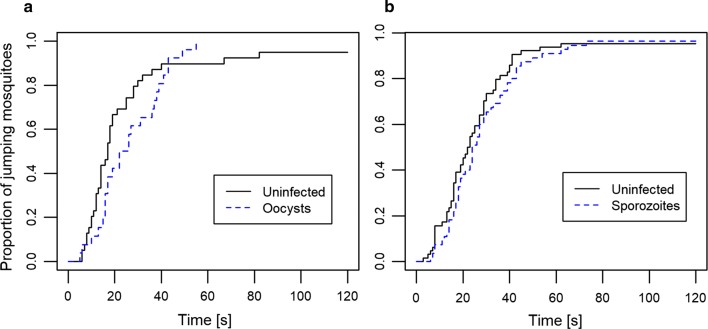
Cumulative proportion of jumping mosquitoes as a function of time since exposure. **a** Proportion measured 11 days after blood feeding, when infected mosquitoes were harbouring oocysts. **b** Proportion measured 21 days after infection, when infected mosquitoes were carrying sporozoites in their salivary glands

Eleven days after infection irritancy was measured for 26 oocyst-infected and 39 uninfected mosquitoes; 21 days after infection it was measured for 52 sporozoite-infected mosquitoes and 64 uninfected ones. On average, it took about 24.3 s for mosquitoes to jump off of the insecticide-treated paper (Fig. 1), and they jumped about 4.8 times per minute. Neither the time up to the first jump (χ^2^ = 1.73, df = 1, p = 0.19) nor the jumping rate (χ^2^ = 1.08, df = 1, p = 0.3) differed significantly between infected and uninfected mosquitoes and neither the time up to the first jump (χ^2^ = 1.19, df = 1, p = 0.28) nor the jumping rate (LMM, χ^2^ = 0.08, p = 0.78) was linked to the age of the mosquitoes. The interaction between the infection status and the time after infection had no significant effect on the time up to the first jump (χ^2^ = 0.01, df = 1, p = 0.93) or the jumping rate (χ^2^ = 0.06, df = 1, p = 0.3). Finally, wing length had no effect on either time up to the first jump (χ^2^ = 0.37, df = 1, p = 0.54) or the jumping rate (χ^2^ = 0.03, df = 1, p = 0.87).

### Blood feeding motivation after exposition to permethrin

None of the mosquitoes died during the trials, i.e., within the 48 h following exposure to the insecticide-treated or untreated paper. The motivation to feed of 329 mosquitoes was analysed. Of the 142 tested 11 days after infection, 62 were infected with oocysts and the remainder were uninfected. Of the 187 tested 21 days after infection, 80 were infected with sporozoites.

**Fig. 2:**
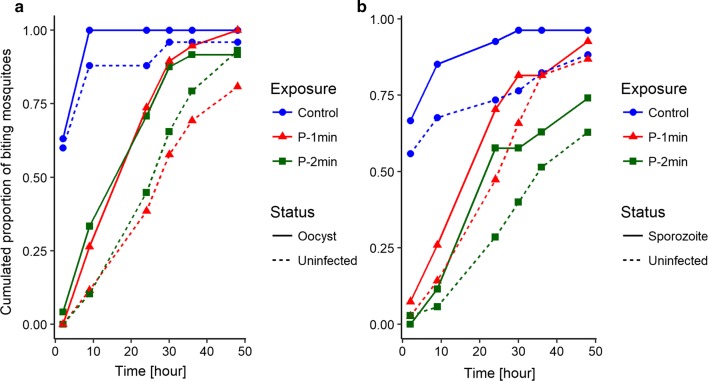
Cumulated proportion of biting mosquitoes in function of time. Colours designate the type of exposure: blue dots denote exposure on control paper; red triangles denote exposure to permethrin for 1 min; green squares denote exposure for 2 min. Continuous lines represent the infected mosquitoes and the dashed lines represent the uninfected ones. **a** Cumulated proportion of biting mosquitoes tested 11 days after infection (i.e., infected with oocysts). **b** Cumulated proportion of biting mosquitoes tested 21 days after infection (i.e., infected with sporozoites)

The summary of the statistical analysis of the proportion of mosquitoes that tried to bite at each test (which, for simplicity, will be called biting rate) is shown in Table 1. The main results are the following. First, if the mosquitoes had been exposed to the control paper, 88.5% of them tried to bite within 24 h after exposure, and their biting rate decreased with time (Fig. 2). If they had been exposed to permethrin, fewer mosquitoes tried to bite within 24 h after exposure (1 min exposure: 57.5%; 2 min exposure: 50.5%) and their biting rate increased with the time.

**Table 1:** Interval count analysis of the motivation to bite of mosquitoes after a previous permethrin or control exposure in function of time

Factors	χ^2^	Df	p-value
Infection status	0.97	1	0.32
Type of exposure	103.24	2	< 0.001
Age	0.01	1	0.92
Time	34.81	5	< 0.001
Infection status: time	11.8	5	0.04
Type of exposure: age	9.1	2	0.01
Type of exposure: time	99.16	10	< 0.001
Age: time	14.16	5	0.01

Second, infected mosquitoes tried to bite earlier than uninfected mosquitoes. Thus, 11 days after infection 81.5% of the oocyst-infected mosquitoes tried to bite within 24 h after being exposed to permethrin, while only 57.1% of the uninfected ones did, and 21 days after infection 73.6% of the sporozoite-infected mosquitoes tried to bite 24 h after exposure while only 49.8% of the uninfected ones did. The statistical analysis (Table 1) confirms that the biting rate decreased more rapidly with time for uninfected mosquitoes than for infected ones. This difference, however, was not affected by the age of the mosquito (so, by the stage of the parasite) (infection status * age * time: χ^2^ = 0.2, df = 5, p = 0.65) or by the type of exposure (infection status * exposure * time: χ^2^ = 5.98, df = 12, p = 0.91). (Note that these two interactions were not included in the final model shown in Table 1).

Third, the young mosquitoes (11 days after their blood meal) attempted to bite earlier than old mosquitoes (21 days after their first blood meal): 24 h post exposition, 69.3% of the young mosquitoes had tried to bite while 61.7% of the old ones had. In addition, the effect of the exposure to permethrin was stronger for old mosquitoes than for young ones. Thus, the time it took to young mosquitoes to try to bite was similar whether they had been exposed to permethrin for 1 or for 2 min (Fig. 2a). In the older mosquitoes, however, the mosquitoes exposed to permethrin for 1 min attempted to bite earlier than those exposed for 2 min (Fig. 2b).

## Discussion

One of the ways ITNs protect their users is that the insecticide irritates mosquitoes when they touch it, so that they are less likely to pass through the net to bite. Since mosquitoes can only be irritated if they are exposed to some insecticide, irritancy can lead to sub-lethal effects of insecticide. Indeed, in this experiment a short exposure to permethrin made mosquitoes less likely to try to bite for almost 48 h without inducing any mortality. While irritancy was not affected by the infection by the malaria parasite, the inhibition of biting was shorter for malaria-infected mosquitoes than for uninfected ones.

The intensity or duration of sub-lethal exposure for mosquitoes depends mainly on how strongly they are irritated. Here the irritancy (measured in two ways: time until first jump and jumping rate [38]) was similar for malaria-infected and for uninfected mosquitoes. This suggests that when facing an ITN, infected and uninfected mosquitoes would receive the same sub-lethal dose of insecticide. It should, however, be noted that the design used here did not allow to measure the persistence of mosquitoes that try to bite through an ITN, which would give a more complete idea about the exposure to the insecticide.

Sub-lethal exposure to insecticides affects the life history traits of insects in various ways [6, 7], including that pyrethroids affects their feeding capacities [18–23]. This study corroborated these results by showing that a short exposure to permethrin (which induced no mortality) alters the mosquito’s host-seeking behaviour. Indeed, the feeding motivation of mosquitoes exposed to permethrin for 1 or 2 min was inhibited for almost 48 h.

More surprising is that this inhibition was weaker for malaria-infected mosquitoes than for uninfected ones. Indeed, our original idea was that infectious mosquitoes (so, those with sporozoites) would be less inhibited and start biting earlier than uninfected ones, for they are more motivated to bite than uninfected or oocyst-infected (and thus non-infectious) ones [26–30]. However, that oocyst-infected mosquitoes were also less inhibited than uninfected ones does not support this idea. Indeed, the manipulation by oocysts (non-infectious stage) should go in the opposite direction: to increase its transmission, the parasite should preserve mosquitoes from host defence-associated mortality, and therefore decrease their motivation to bite [28].

An alternative explanation for the weaker effect of permethrin on infected mosquitoes may be linked to oxidative stress. Indeed, the production of reactive oxygen species (ROS) is increased by both infection by malaria [45–48] and pyrethroid exposure [49, 50]. After an infected blood meal, mosquitoes modulate this ROS production by expressing a higher level of antioxidant enzymes in the fat body [45]. Similarly, survival to pyrethroid exposure requires sufficient activity of anti-oxidant enzymes to avoid lipid peroxidation and cell damage [51]. It is therefore possible that the higher anti-oxidant defence triggered by an infection with *Plasmodium* may, to some extent, help to deal with a further exposure to a pyrethroid insecticide. Furthermore, detoxification mechanisms may also play a role. It is known for example that following infection by *Plasmodium*, the cytochrome P450 CYP6M2, an enzyme involved in pyrethroid’s detoxification [52, 53], is upregulated [54]. Thus, because of the higher production of both anti-oxidant and cytochrome P450 enzymes, it is not worth to expect infected mosquitoes to recover faster after permethrin exposure. This could help to explain why, in this study, they started to probe sooner after exposure. Such a link would have epidemiological implications, for it implies that infected mosquitoes would be more tolerant to pyrethroids. Glunt and coworkers [55] investigated this link, and reported no effect of *P. yoelii* sporozoite-infection on *An. stephensi* resistance to permethrin, but slightly higher knockdown resistance for mosquitoes that had fed on infectious blood than for those fed on uninfected blood. However, as they mentioned, some of the mosquitoes that had fed on infectious blood may not have been infected, possibly hiding any greater effect of malaria infection on permethrin sensitivity.

A further look into the different mechanisms implied in pyrethroid metabolism may also help to explain the longer inhibition of blood feeding behaviour in older mosquitoes. A possibility may be linked to the fact that resistance to insecticides often decreases with age (e.g., [34, 56, 57]), which is due in part to the age-related decline of the activity of P450 mono-oxygenases [58], esterases [59] and glutathione-*S*-transferases [56], the three main enzymatic systems responsible for pyrethroid resistance in mosquitoes [60, 61]. Old mosquitoes may thus need more time to detoxify and recover after a pyrethroid exposure. This idea would be consistent with the observation of this study: that old mosquitoes responded differently to the duration of exposure (1 or 2 min), whereas young mosquitoes showed a similar inhibition response for the two exposures.

Whatever the mechanisms, the additional inhibition of mosquito’s host-seeking behaviour induced by permethrin may have strong implications for the epidemiology of malaria. Indeed, each time a mosquito is exposed to a sub-lethal dose of permethrin, it will postpone its blood meal and thus might lengthen its gonotrophic cycle [45]. This would strongly affect not only the mosquito’s reproductive success, but also the transmission of malaria. First, lower biting rate of uninfected mosquitoes further than what is expected from the physical barrier and repellency of ITNs would further decrease the rate of encountering malaria. Second, the lower biting rate of oocyst-infected mosquitoes would increase the chance that oocyst-infected mosquitoes would survive to harbour sporozoites. Third, the inhibition of the infectious mosquitoes would reduce the rate at which sporozoites are transmitted. Since, however, that the infection reduces the inhibition of biting, it also reduces the impact of the sub-lethal exposure on the epidemiology of malaria through the two latter effects.

Whether such effects are indeed observed depends on whether we see similar patterns in a natural system. Furthermore, since some effects of inhibition can increase transmission while others decrease it, these results should be confirmed not only qualitatively, but also quantitatively with a natural human malaria parasite in different epidemiological settings.

## Conclusions

ITNs offer personal protection in part by irritating mosquitoes and thereby reducing the probability to being bitten by an infectious mosquito. Here, irritancy had an additional effect than just reducing the time mosquitoes spend on the net trying to bite or pass through it. Indeed, sub-lethal exposure (1 or 2 min) to permethrin, inhibited the host-seeking behaviour of mosquitoes by impeding them to blood feed for almost 48 h, confirming the results of previous studies [22, 23]. In addition, the results of this study complement a study showing that the sporozoites of malaria reduce the repellency of an insecticide [31]. Thus, malaria infection shortened the inhibition caused by the insecticide at, both, its developmental (oocyst) and infectious (sporozoite) stages. The results of this study may help to get a better measure of the impact of ITNs for malaria control. On the one hand, the inhibition of the mosquito’s host-seeking behaviour might reduce the chance they encounter and/or transmit malaria, thus increasing not only the protection of the ITNs users, but also the protection of the non-users. On the other hand, by shortening the inhibition of mosquito feeding behaviour, both oocysts and sporozoites might weaken both personal and community protections.

## Additional file

**Additional file 1: Table S1.** Dataset Irritancy. Data used for statistical analysis of irritancy. **Table S2.** Dataset Motivation to bite. Data used for statistical analysis of the motivation of mosquitoes to bite after permethrin exposure (interval count analysis).
